# Overexpression of Glutathione Transferase E7 in Drosophila Differentially Impacts Toxicity of Organic Isothiocyanates in Males and Females

**DOI:** 10.1371/journal.pone.0110103

**Published:** 2014-10-16

**Authors:** Aslam M. A. Mazari, Olle Dahlberg, Bengt Mannervik, Mattias Mannervik

**Affiliations:** 1 Department of Neurochemistry, The Wenner-Gren Institute, Stockholm University, Stockholm, Sweden; 2 Department of Molecular Biosciences, The Wenner-Gren Institute, Stockholm University, Stockholm, Sweden; Alexander Fleming Biomedical Sciences Research Center, Greece

## Abstract

Organic isothiocyanates (ITCs) are allelochemicals produced by plants in order to combat insects and other herbivores. The compounds are toxic electrophiles that can be inactivated and conjugated with intracellular glutathione in reactions catalyzed by glutathione transferases (GSTs). The *Drosophila melanogaster* GSTE7 was heterologously expressed in *Escherichia coli* and purified for functional studies. The enzyme showed high catalytic activity with various isothiocyanates including phenethyl isothiocyanate (PEITC) and allyl isothiocyanate (AITC), which in millimolar dietary concentrations conferred toxicity to adult *D. melanogaster* leading to death or a shortened life-span of the flies. *In situ* hybridization revealed a maternal contribution of GSTE7 transcripts to embryos, and strongest zygotic expression in the digestive tract. Transgenesis involving the GSTE7 gene controlled by an actin promoter produced viable flies expressing the GSTE7 transcript ubiquitously. Transgenic females show a significantly increased survival when subjected to the same PEITC treatment as the wild-type flies. By contrast, transgenic male flies show a significantly lower survival rate. Oviposition activity was enhanced in transgenic flies. The effect was significant in transgenic females reared in the absence of ITCs as well as in the presence of 0.15 mM PEITC or 1 mM AITC. Thus the GSTE7 transgene elicits responses to exposure to ITC allelochemicals which differentially affect life-span and fecundity of male and female flies.

## Introduction

Organic isothiocyanates (ITCs) are reactive biomolecules that occur in plants, particularly abundantly in cruciferous species. The compounds are predominantly stored as unreactive glucosinolates from which they are released by hydrolysis catalyzed by the enzyme myrosinase [1]. ITCs are regarded as important contributors to the protection of plants from attacks by insects and microorganisms, and the release of the compounds is triggered by insults to the plant tissue. The interplay between plants and the offending insects have obviously evolved such that the emergence of insecticidal compounds has been countered by the evolution of detoxication enzymes in insects. For example, glucosinolates of brassicaceous plants stimulate oviposition of specialist insects such as *Pieridae* butterflies, and the caterpillars feeding on the plants resist the ITC toxicity [2] Studies of *Arabidopsis thaliana*, a plant displaying glucosinolates as their primary defensive trait, demonstrate that six different ITC chemotypes are present in different proportions in separate geographical populations [3]. The *A. thaliana* ITC chemotype was altered in five generations in response to experimental exposure to different herbivorous aphids feeding on the plants.

ITCs are strong electrophiles that exert toxicity by reacting with sulfhydryl groups and other nucleophilic chemical residues in biological tissues. The most abundant low-molecular cellular thiol nucleophile is glutathione, which inactivates ITCs by the formation of dithiocarbamates [4]. This reaction is efficiently catalyzed by many glutathione transferases (GSTs) [5], and it has been proposed that feeding on mustard plants and ITC activity of GSTs has coevolved [6]. It would appear likely that insect GSTs provide protection against ITC toxicity, but this notion has not been experimentally tested. In the present study we have created a transgenic *Drosophila melanogaster* overexpressing the enzyme GSTE7, which is shown to be highly active with ITC substrates *in vitro*, and studied the effect of allyl isothiocyanate (AITC) and phenethyl isothiocyanate (PEITC) on the transgenic flies.

## Materials and Methods

### Synthesis of DNA encoding GSTE7 and plasmid for heterologous expression of the enzyme

The GSTE7 open reading frame of *Drosophila melanogaster* gene CG17531 was custom synthesized by DNA 2.0, Inc., Menlo Park, CA, and provided in the pJ201 plasmid as kind gift by Dr. Claes Gustafsson. For heterologous expression of the GSTE7 protein a synonomous nucleotide sequence with a codon usage optimized for *Escherichia coli* was synthesized and a His_6_-tag introduced at the N-terminus. The optimized nucleotide sequence was provided in the pJexpress401 expression vector and used for transformation of electroporation-competent *E. coli* XL-1 Blue cells by electroporation. Briefly, 2 µl of plasmid DNA (17 ng/µl) and 48 µl of *E. coli* cells were gently mixed and placed in a Gene pulser cuvette with 0.1 cm electrode gap (Bio-Rad). After an electric pulse of 4.5 s at a voltage of 1.25 kV, 960 µl of LB medium was added followed by gentle mixing by repeated pipetting. Finally, the cells were incubated at 37°C in a 10 ml tube containg LB medium and agitated at 200 rpm for 45 min. After incubation, the cells were spread on LB agar plates containing 50 µg/ml of kanamycin and incubated for 16 h at 37°C.

### Protein expression and purification

A starter culture of 50 ml of 2×YT medium containing 50 µg/ml of kanamycin was inoculated with a single colony of freshly transformed cells and incubated for 16 h with shaking at 200 rpm at 37°C. After incubation, 5 ml starter culture was taken to inoculate 500 ml of 2×YT medium containing 50 µg/ml kanamycin. The inoculated cells were incubated at 30°C in an incubate-shaker at 200 rpm till the required OD_600_ 0.46 was obtained. Subsequently the GST expression was induced with 0.1 mM isopropyl-β-D-thiogalactopyranoside (IPTG). After incubation for 16 h at 37°C, the cells were harvested at 7000 rpm for 10 min at 4°C (Avanti J-20 XP Beckman Coulter USA). The supernatant was discarded and the pellet containing bacteria was resuspended in 30 ml of ice-cold buffer A (20 mM sodium phosphate pH 7.4, containing 85 mM imidazole, 500 mM NaCl, 10 mM β-mercaptoethanol and 0.02% sodium azide) and 0.2 mg/ml of lysozyme and one tablet of EDTA-free protease inhibitor (Roche Germany). After incubation for 30 min on an ice bath, the cells were lysed by sonication (Vibra cell USA) for 5×20 s at an output control of 7.5 with an interval of 1 min on an ice bath to avoid heating the sample. The resultant suspension was centrifuged at 27200 g for 1 h at 4°C. The pellet was discarded and the supernatant containing the protein was incubated with pre-equilibrated Ni-IMAC gel on an ice bath for 30 min. After incubation, the gel was washed extensively with milli-Q water and packed into a column. The unbound proteins were further washed away by buffer A and the bound protein eluted with buffer B (20 mM sodium phosphate pH 7.4, containing 500 mM imidazole, 500 mM NaCl, 10 mM β-mercaptoethanol and 0.02% sodium azide). The eluted fractions were pooled and dialyzed overnight against 10 mM Tris HCl buffer pH 7.8, containing 0.2 mM DTT and 1 mM EDTA. The protein was concentrated and the concentration was measured by Bradford Standard Assay (Bio-Rad USA). The homogeneity and the purity of the enzyme was confirmed by SDS-PAGE analysis, using a 12.5% (w/v) polyacrylamide resolving gel [7]. The purified enzyme was stored in aliquots at −80°C.

### Enzyme activity assays of GSTE7

1-Chloro-2,4-dinitrobenzene (CDNB) was used as standard substrate for monitoring the enzyme purification. The reaction was measured at 30°C by the increase in absorbance (Δε_340nm_ = 9600 M^−1^cm^−1^) accompanying GSH conjugation (Table 1). For screening assays allyl isothiocyanate (AITC) and phenethyl isothiocyanate (PEITC) were also included (Table 1). Reaction with the ITC substrates was detected at 274 nm with a Δε_274nm_ at 7450 M^−1^cm^−1^ and 8890 M^−1^cm^−1^ for AITC and PEITC, respectively [5].

**Table 1:** Assay conditions for the specific activity determination with alternative substrates.

Substrate	GSH(mM)	Substrate (mM)	Wavelength(nm)	Δ 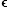 (mM^−1^ cm)	pH
Allyl isothiocyanate	1	0.4	274	7.45	6.5
Benzyl isothiocyanate	1	0.4	274	9.25	6.5
Propyl isothiocyanate	1	0.4	274	8.35	6.5
Phenethyl isothiocyanate	1	0.4	274	8.89	6.5
Sulforaphane	1	0.4	274	8.00	6.5
1-chloro-2,4-dinitrobenzene	1	1	340	9.6	6.5

All measurements were performed in 0.1 M sodium phosphate buffer, pH 6.5 at 30°C. The stock solutions for Isothiocyanates were prepared in acetonitrile (2% final concentration in the assay), and that for CDNB in ethanol (5% final concentration in the assay).

### In situ hybridization

The GSTE7 coding sequence was PCR amplified from the pJ201 plasmid with the forward and reverse primers CTCGGGATCCATGCCCAAATTGAT and ATCGAAGCTTATTCGATGCGAAAGTG, and TA-cloned into pGEM-T Easy (Promega). The resulting plasmid was linearized with Apa I and a digoxigenin-labeled RNA probe transcribed with SP6 RNA polymerase. Whole mount *in situ* hybridization of *Drosophila* embryos with the GSTE7 probe was performed as previously described [8].

### Construction of transgenic flies

The coding sequence of GSTE7 in the pJ201 plasmid was inserted into the pUAST attB vector [9] with Not I and Xba I. Embryos from *y^1^ sc^1^ v^1^ P{nos-phiC31\int.NLS}X; P{CaryP}attP2* flies containing the attP2 landing site on chromosome 3L (68A4) were injected with the plasmid by Rainbow Transgenic Flies, Inc., Camarillo, CA, USA.

### Survival and egg-laying assays

Approximately 100 *Actin-Gal4*/*CyO* virgin females were crossed to 40 *w^1118^* (control) or to 40 *UAS*-*GSTE7* homozygous males and kept in food bottles. Offspring from the crosses were allowed to mate with each other for 3–5 days after hatching in the bottles. Male and female 3–5 days old non-*Cy* flies were then divided into separate vials at the first day of the assays. Flies were transferred to new vials 3 times per week during the survival assay. Oviposition was measured on seven consecutive days. The flies were transferred to fresh vials with or without ITC each day, and the number of eggs deposited in the vials counted.

One liter of food was prepared by adding 12.9 g of yeast, 40 g instant mashed potatoes, and 10 g of agar in 1.1 liter of boiling water, and 50 ml of syrup added when the mixture was homogeneous. After boiling for 20 min, the mixture was cooled to 55°C before being supplemented with 0.875 g ascorbic acid, 8.5 ml 10% Nipagin in ethanol, 6.25 ml propionic acid, and 10 ml of 99% ethanol (control) or isothiocyanate compound dissolved in 10 ml of 99% ethanol. Ten males or 10 females were placed in each vial and kept at 25°C.

Furthermore, we tested for any food avoidance behavior induced by the addition of PEITC. For this, we used xylene cyanol in fly food prepared as above. We monitored the uptake of food by means of flies turning blue. Males and females were separated and put in food vials (5 flies/vial). After three hours in food vials, all flies of both sexes had a blue colored abdomen, and no differences between flies given control food and drug supplemented food could be seen.

## Results

### Enzymatic activities of Drosophila melanogaster GSTE7

The purified GSTE7 was functionally characterized by enzymatic assays with alternative substrates (Table 2). In order to test general GST activity, the standard substrate 1-chloro-2,4-dinitrobenzene (CDNB) was used. The specific activity of 36.5 µmol·min^−1^·mg^−1^ determined is within the common range of efficient GSTs, demonstrating that GSTE7 is catalytically highly competent. Further, a series of organic isothiocyanates (ITCs) of biological significance were investigated as substrates. These compounds are plant allelochemicals and include ITCs that feature both aliphatic and aromatic chemical substituents. The specific ITC activities were of similar magnitude and ranged between 7.5 and 23.8 µmol·min^−1^·mg^−1^ (Table 2). These values are approaching the specific ITC activities of the most active human GSTs [5] and suggest that *D. melanogaster* expressing GSTE7 are highly competent to conjugate and inactivate ITCs when the flies are exposed to these electrophilic toxicants. For our *in vivo* experiments the demonstration of high enzyme activities with allyl-ITC (AITC) and phenethyl-ITC (PEITC) are particularly relevant.

**Table 2:** Specific activities of purified GSTE7 with alternative substrates.

Substrate	Specific activity (µmol/min per mg of protein)
1-chloro-2,4-dinitrobenzene (CDNB)	36.5±1.12
Allyl isothiocyanate	11.2±0.48
Benzyl isothiocyanate	16.0±0.76
Propyl isothiocyanate	7.9±0.49
Phenethyl isothiocyanate	7.5±1.37
Sulforaphane	23.8±1.08

The data are means ± SD of 3 replicate measurements and the background activities were corrected by using the same concentration of solvent without enzyme.

### Overexpression of D. melanogaster GSTE7

**Figure 1:**
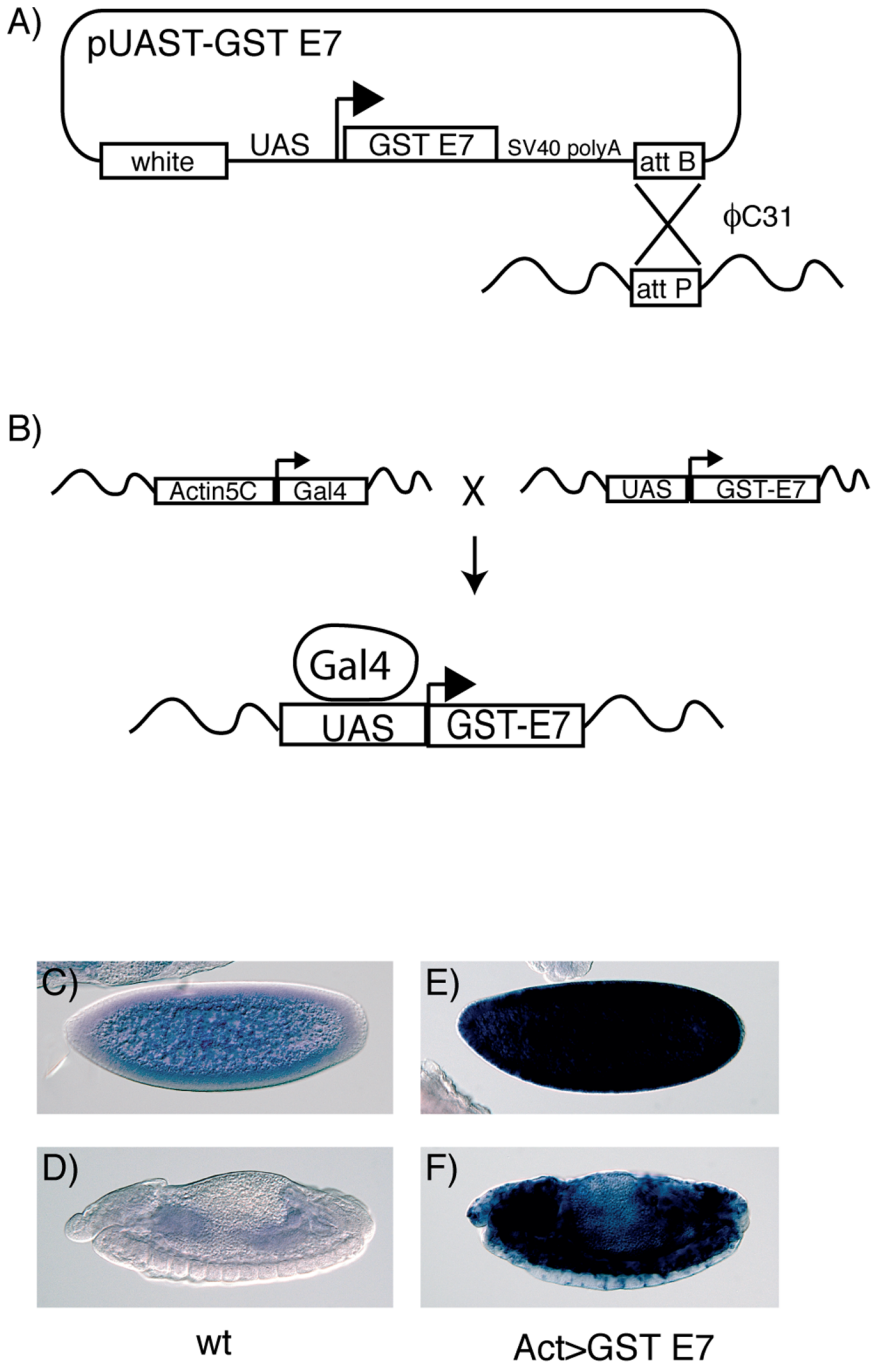
Overexpression of the GSTE7 protein in fruit flies (*Drosophila melanogaster*). (A) Scheme outlining the binary Gal4-UAS system [10] used to induce ubiquitous overexpression. The coding sequence of the *Drosophila* GSTE7 gene was inserted in the pUAST attB vector [9] containing an upstream activating sequence (UAS) and a downstream attB crossover site. The plasmid was introduced by injection into fly embryos carrying an attP landing site on chromosome 3 and inserted into the chromosome by way of ΦC31-mediated integration. (B) Flies overexpressing the GSTE7 transgene were obtained by crossing the UAS-GSTE7 flies with a strain bearing the Actin-Gal4 driver, which induces ubiquitous expression. (C–F) Embryonal expression of *D. melanogaster* GSTE7 detected by *in situ* mRNA hybridization. Fly embryos were stained with a digoxigenin-labeled probe recognizing GSTE7 by whole-mount *in situ* hybridization, and are oriented with anterior to the left and dorsal up. (C) Noninduced pre-cellular embryo showing endogenous GSTE7 mRNA, which is maternally contributed and therefore distributed ubiquitously in the embryo. (D) Stage 13 embryo showing GSTE7 expression lower than in the early embryo, but with pronounced staining in the midgut. In the presence of the Actin-Gal4 driver, the transgenic tissues displayed high ubiquitous expression of GSTE7 in both pre-cellular (E) and stage 13 (F) embryos.

To investigate the *in vivo* effects of elevated GST levels, we took advantage of the binary Gal4-UAS system to induce overexpression of GSTE7 protein in *Drosophila* [10]. We cloned the coding sequence of the *Drosophila* GSTE7 gene together with an upstream activating sequence (UAS) and introduced the construct on chromosome 3 carrying an attP landing site by way of phiC31-mediated integration (Fig. 1A) [9].

The ubiquitous Actin-Gal4 driver was crossed to UAS-GSTE7 flies to induce expression from the transgene (Fig. 1B). To confirm the overexpression, embryos were stained with a probe recognizing GSTE7 mRNA by whole-mount *in situ* hybridization (Fig. 1C–F). As shown in Fig. 1C, endogenous GSTE7 mRNA is generally distributed throughout pre-cellular embryos prior to zygotic transcription, demonstrating a maternal contribution. In stage 13 embryos, GSTE7 expression is considerably weaker, but most pronounced in the midgut (Fig. 1D). In the presence of the Actin-Gal4 driver, we observed high-level expression of GSTE7 throughout both pre-cellular and stage 13 embryos (Fig. 1E and F). This shows that transgenic GSTE7 is overproduced both in the female germline and in somatic tissues. Expression of the transgene did not diminish survival, suggesting that the GSTE7 transgene by itself did not cause any harmful physiological effects.

### Impact of transgenic GSTE7 expression on PEITC exposure

**Figure 2:**
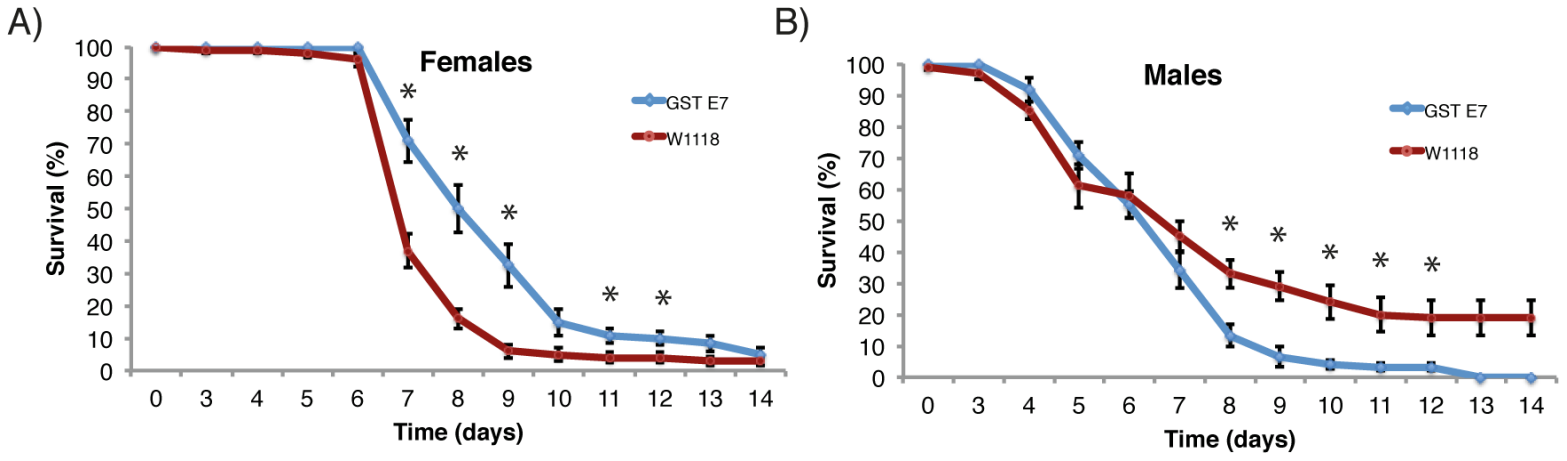
Survival of adult flies on 0.25 mM PEITC added to standard fly food. Actin-Gal4 flies crossed to wild-type control (*w^1118^*) or to UAS-GSTE7 flies (GSTE7) were allowed to mate and then transferred to fly food supplemented with PEITC. Twelve vials with 10 flies each (GSTE7) or 10 vials with 10 flies each (*w^1118^*) were used and the number of surviving flies in each vial scored. Females (A) and males (B) were kept in separate vials. Error bars represent standard error of the mean. * indicates P<0.05, two-tailed unpaired Student's t-test.

**Figure 3:**
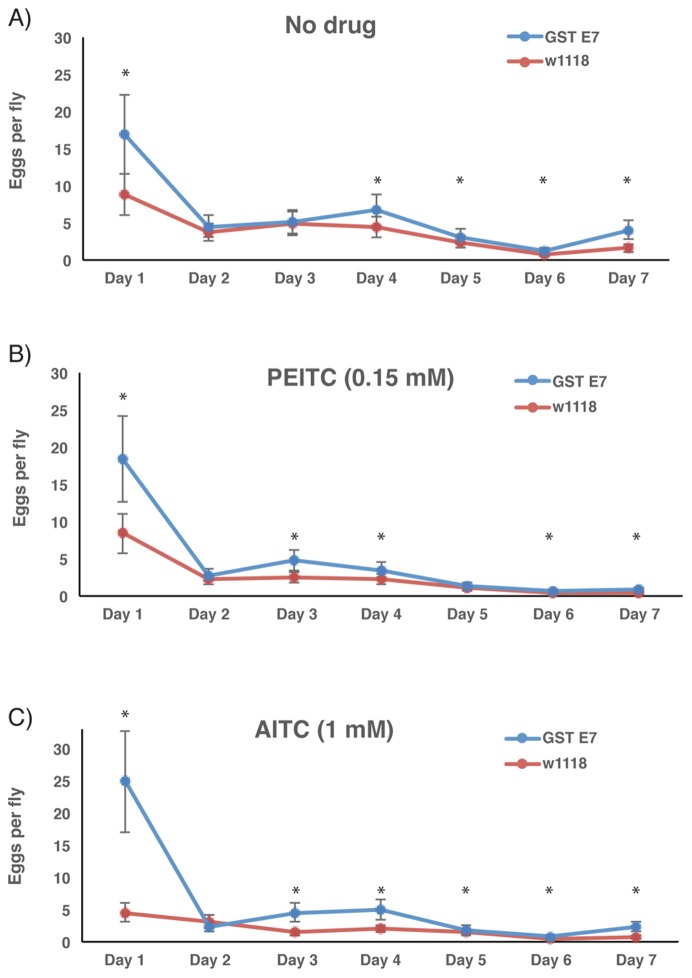
Effect of ITCs on egg laying of fruit flies. Actin-Gal4 flies crossed to wild-type control (*w^1118^*) or to UAS-GSTE7 flies (GSTE7) were mated for 3–5 days and the females separated and then kept for 7 days on standard control, 0.15 mM PEITC, or 1 mM allyl-ITC supplemented food. They were transferred to fresh vials each day and the number of eggs in the vials counted. Ten vials (approximately 10 flies/vial) with control (*w^1118^*) or GSTE7 flies were used and the number of eggs divided by the number of flies. The graph shows the number of eggs laid per fly, and error bars represent standard error of the mean. * indicates P<0.05, two-tailed unpaired Student's t-test.

We tested the *in vivo* effects of PEITC, an efficient substrate of GSTE7, in our *in vitro* assay. A concentration of 0.25 mM PEITC in standard fly food was shown to be toxic and significantly shortened the lifespan of wild-type flies, and higher PEITC concentrations were fatally toxic. We noticed that overexpression of GSTE7 could protect females from the toxic effects of 0.25 mM PEITC during days 7–12 of exposure, but had no positive effect on long-term survival (Fig. 2A). By contrast, the effect on males was the opposite to that on females, such that a significantly higher mortality was seen in fly males overexpressing GSTE7 after one week of exposure (Fig. 3B). A concentration of 4 mM AITC in the standard fly food had a lethal effect on both wild-type and transgenic flies, resulting in death in a few hours after first exposure, but subjected to 1 mM AITC in the diet, flies and larvae were viable and showed no obvious weakness or other phenotype.

### Effect of ITCs on oviposition

As a corollary to the differential effects of the transgene on males and females, the *in vivo* effects of AITC and PEITC, demonstrated to be effective GSTE7 substrates in our *in vitro* studies (Table 2), were investigated with respect to oviposition. The number of eggs produced by 3–5 days old flies were counted for seven consecutive days, which demonstrated a significant influence of the transgene on the rate of oviposition (Figure 3). Unexpectedly, the effect was demonstrated both in the presence and the absence of ITCs. The number of eggs was increased about twofold in the transgenic flies on day 1 as compared to the wild-type flies, and consistently showed a higher oviposition rate than the wild-type flies both in the absence and presence of ITCs (Fig. 3). This effect of the transgene was statistically significant on most days of the assay. Exposure to AITC or PEITC in the diet demonstrated an approximately 5- to 2-fold increase in egg-laying ability in the transgenic flies expressing GSTE7 in comparison with that of wild-type flies.

## Discussion

For half a century a chemical warfare of plants against herbivorous insects has been viewed as a major driver of co-evolution of the combatants [11]. In *Brassica* plants the chemical weapons are largely based on glucosinolates that decompose into ITCs and other toxic products when plant tissues are crushed or when they are digested by the feeding insects. The detoxication of the released ITCs has not been studied in great detail, but it is generally assumed that GSTs play a pivotal role in the biotransformation of ITCs.

Differences among insects may occur, but recent studies of lepidopteran caterpillars feeding on glucosinolate-containing plants show that a major fraction of the corresponding ITC metabolites are excreted in the feces as products of glutathione conjugation [12]. Two strains of the whitefly *Bemisia tabaci*, biotype B and biotype Q, feeding on *Arabidopsis thaliana* plants showed different responses to their host plants suggesting evolutionary divergence [13]. *A. thaliana* is known to contain at least three dozen glucosinolates distinguishable by their sidechains that can be aliphatic or aromatic, and the whiteflies were exposed to transgenic *A. thaliana* plants that overproduced either aliphatic or aromatic (indole-containing) glucosinolates. Significant differences in the behavioral and biochemical responses were noted between whiteflies of biotypes B and Q, but both biotypes responded by a reduced number of oviposited eggs when reared on transgenic plants overproducing glucosinolates. Biotype Q displayed higher constitutive levels than biotype B of several detoxication enzymes and two GSTs were significantly induced during exposure to *A. thaliana* featuring overproduction of indole-containing glucosinolates, suggesting a role of GSTs in detoxication and an explanation of the good performance on the indole-glucosinolate accumulating plants [13].

Our experiments establish overexpression of GSTE7 in *D. melanogaster* without detectable behavioral or morphological alterations of the phenotype. However, GSTE7 overexpression had a significant effect on oviposition (Fig. 3). Since elevated levels were present both in the germline, as shown by enhanced transcript levels in embryos prior to zygotic transcription (Fig. 1), and in somatic tissues, GSTE7 could be influencing oogenesis either directly or indirectly.

AITC and PEITC are produced in plants and are known toxins that may have influenced the evolutionary interplay between *Drosophila* species and *Brassica* plants [14], [15]. GSTs are catalyzing the inactivation of these compounds as well as other ITCs that have been investigated [16]. GSTE7 overexpression provided significant protection of female *D. melanogaster* against ITCs represented by PEITC in Fig. 2. For unclear reasons, no significant protection of males was noted. Instead, the transgene significantly reduced the survival rate of males. The explanation of this deleterious influence is not clear, but excessive ITC conjugation may lead to glutathione depletion and toxicity or apoptosis [17]. Glutathione is involved in a multitude of reactions and conjugation may shift the tissue redox balance, which is known to influence longevity.

ITCs are not only directly toxic but also act as repellants of certain insects. Studies of *Drosophila* demonstrate that isothiocyanates in wasabi (including AITC) lead to a food avoidance behavior involving the transient receptor potential A1 gene *painless* in gustatory receptor neurons [18], [19]. However, the detrimental effects of ITCs in our experiments cannot be explained primarily by food avoidance and starvation, since the GSTE7 transgene provided protection. Furthermore, to check for food avoidance behavior xylene cyanol was added to the diet, which results in a blue abdomen when consumed, but no difference in coloring was noted between flies fed ITC-containing or control food.

Canonical GSTs occur in *D. melanogaster* in multiple forms, the GSTome, encoded by 36 different genes in six distinct classes and the enzymes have previously been subjected to preliminary enzymological studies [20], [21]. GSTs from the Delta (D) and Epsilon (E) classes have the largest number of members and are those primarily associated with the defense against toxicants. GSTE7 is one of a limited number of GSTs that are overexpressed in long-lived *D. melanogaster* [22], suggesting that it may provide survival value to flies. This enzyme was therefore selected for biochemical characterization and transgenesis studies. Individual GSTs often have overlapping substrate acceptance, and gene disruption studies may consequently not reveal the importance of individual genes, whereas overexpression of a particular GST from a corresponding transgene could be more informative.

Examination of embryos of different ages demonstrates that the GSTE7 transcript has a general distribution with particularly high concentration in the digestive tract (Fig. 1). Furthermore, the gut of larvae and adult flies also show high expression of GSTE7 according to the FlyAtlas Anatomical Expression Data [23], suggesting that the normal function of GSTE7 could involve metabolism of ITCs and other toxic electrophiles originating from food and liquid uptake. In summary, it would appear that flies exposed to ITCs at the first level of protection rely on avoidance mechanisms based on gustatory receptors [18], [19]. However, any ITCs ingested will at the second level undergo chemical inactivation catalyzed by GSTs leading to conjugates suitable for excretion. Even though reactions catalyzed by GSTs are instrumental in the biotransformation of ITCs and other toxic electrophiles, excessive conjugation activity may lead to glutathione depletion and be detrimental, as suggested by the data obtained with the male flies (Fig. 2B). Obviously, the cellular processes have to be properly regulated for optimal adaptation to ambient life conditions.
